# Why does decreased likeability not deter adolescent bullying perpetrators?

**DOI:** 10.1002/ab.21824

**Published:** 2019-02-01

**Authors:** Claire F. Garandeau, Tessa A. M. Lansu

**Affiliations:** ^1^ Research Centre Adolescent Development Utrecht University Utrecht The Netherlands; ^2^ Department of Psychology and Speech‐Language Pathology, University of Turku Turku Finland; ^3^ Behavioural Science Institute, Radboud University Nijmegen The Netherlands

**Keywords:** aggression, bullying, likeability, peer status, popularity, social cognition

## Abstract

This study examines why the lower likeability of bullying perpetrators does not deter them from engaging in bullying behavior, by testing three hypotheses: (a) bullying perpetrators are unaware that they are disliked, (b) they value popularity more than they value likeability, (c) they think that they have nothing to lose in terms of likeability, as they believe that their targets and other classmates would dislike them anyway, regardless of their behavior. The first two hypotheses were examined in Study 1 (1,035 Dutch adolescents, *M*_age_ = 14.15) and the third hypothesis was examined in Study 2 (601 Dutch adolescents, *M*_age_ = 12.92). Results from regression analyses showed that those higher in bullying were not more likely to overestimate their likeability. However, they were more likely than others to find being popular more important than being liked. Moreover, those higher in bullying were more likely to endorse the belief that the victimized student or the other classmates would have disliked a bullying protagonist (in vignettes of hypothetical bullying incidents) before any bullying started. These findings suggest that adolescent bullying perpetrators may not be deterred by the costs of bullying in terms of likeability, possibly because they do not value likeability that much (Hypothesis 2), and because they believe they hardly have any likeability to lose (Hypothesis 3).

## 1 INTRODUCTION

The recognition of school bullying as a pervasive and harmful phenomenon has given rise to the development of numerous antibullying interventions in the last three decades. However, even successful programs have failed to produce large declines in bullying behavior (Jiménez‐Barbero, Ruiz‐Hernàndez, Llor‐Zaragoza, Pérez‐García, & Llor‐Esteban, 2015; Ttofi & Farrington, 2011) and their effectiveness may be limited to bullying perpetrators who are not highly popular (Garandeau, Lee, & Salmivalli, 2014) and to childhood or early adolescence (Jiménez‐Barbero et al., 2015; Yeager, Fong, Lee, & Espelage, 2015). Many researchers now concur that school bullying—which is repeated, proactive aggression—is not easily reduced because it rewards those engaging in it with high peer status (Garandeau et al., 2014; Volk, Camilleri, Dane, & Marini, 2012). In fact, new antibullying strategies, such as the meaningful roles intervention, focus on providing bullying perpetrators with means to achieve high popularity through prosocial rather than aggressive means (Ellis, Volk, Gonzalez, & Embry, 2016). In adolescence, bullying others predicts high levels of perceived popularity (Caravita, Di Blasio, & Salmivalli, 2009; De Bruyn, Cillessen, & Wissink, 2010; Duffy, Penn, Nesdale, & Zimmer‐Gembeck, 2017; Pouwels, Lansu, & Cillessen, 2016; Reijntjes et al., 2013a; Sijtsema, Veenstra, Lindenberg, & Salmivalli, 2009; Vaillancourt, Hymel, & McDougall, 2003; Van den Broek, Deutz, Schoneveld, Burk, & Cillessen, 2016) and gains in social dominance (Reijntjes et al., 2013b). Moreover, those who bully tend to strive for popularity more than others (Caravita & Cillessen, 2012; Sijtsema et al., 2009). Thus, the widespread and continued high prevalence of bullying, which is a goal‐directed behavior (Volk, Dane, & Marini, 2014), may be explained by the high‐status bullying confers on its perpetrators, which is something they aim to obtain. To the extent that it provides access to desired resources, bullying is both rewarding from a social learning perspective (Bandura, 1971) and adaptive from an evolutionary perspective (Volk et al., 2012, 2014).

Nevertheless, bullying others also incurs social costs for the perpetrators: It is negatively associated with social preference (or likeability), indicating that adolescents who bully are generally disliked by their peers (Caravita, Di Blasio, & Salmivalli, 2009; Dijkstra, Lindenberg, & Veenstra, 2008; Pouwels et al., 2016; Sentse, Kiuru, Veenstra, & Salmivalli, 2014; Sijtsema et al., 2009; Vaillancourt et al., 2003; Van den Broek et al., 2016). It remains unclear, however, why this lower likeability does not discourage these adolescents from bullying. It is surprising, as being liked by one’s peers provides individuals with feelings of affection and helps them fulfill their desire for interpersonal attachment, which is a fundamental human need (Baumeister & Leary, 1995; Pendell, 2002). Being liked should, therefore, also be rewarding and adaptive for human beings. Identifying cognitions underlying the lack of responsiveness of bullying perpetrators to the social costs of their behavior could guide future antibullying efforts.

The current study investigates why lower likeability does not act as a deterrent for adolescents engaging in bullying, by putting three possible explanations to the test. First, bullying perpetrators may not be aware of being disliked. In other words, they may overestimate their likeability. We refer to this explanation as the *inaccuracy of self‐perceived likeability* hypothesis. Second, lower likeability may not matter to them because they value being perceived as popular more than they value being liked by their peers, which we refer to as the *superiority of popularity* hypothesis. Third, we considered the possibility that bullying perpetrators might think that they have no likeability to lose anyway. Though they might believe that high perceived popularity is within their reach, they might also believe—rightly or not—that their targets and other classmates would not like them, irrespective of their behavior. In this case, they would only have something to gain by bullying—perceived popularity—and nothing to lose in terms of likeability. We refer to this explanation as the *unreachability of likeability* hypothesis. As the three hypotheses are not mutually exclusive, it is possible that we will find evidence for more than one hypothesis.

### 1.1 The inaccuracy of self‐perceived likeability hypothesis

The first reason why lower likeability does not discourage bullying adolescents from engaging in such behavior may be a lack of awareness of their lower status. Perpetrators who are disliked by peers may believe that they are relatively well‐liked, possibly because they might mistake the fear that they instill in others for a form of respect (Vaillancourt, McDougall, Hymel, & Sunderani, 2010). To our knowledge, no study has examined the link between bullying behavior specifically and overestimation of likeability (or peer acceptance) in adolescents. Nevertheless, several studies examining aggressive behavior in children indicate that positively biased perceptions of peer acceptance are associated with higher levels of both overt and relational aggression (David & Kistner, 2000; Lynch, Kistner, Stephens, & David‐Ferdon, 2016) and general aggression (Sandstrom & Herlan, 2007; Stephens, Lynch, & Kistner, 2016). This positive association was found when biased self‐perceptions of peer acceptance were operationalized as the variance in children’s self‐perceived likeability unexplained by their actual (or peer‐perceived) likeability (David & Kistner, 2000; Lynch et al., 2016) as well as when operationalized as difference scores between actual and self‐perceived likeability (Sandstrom & Herlan, 2007; Stephens et al., 2016).

However, not all studies on aggression and overestimation of likeability provide evidence for a positive link between the two: When children’s reactive and proactive aggression were examined separately and teachers were used as informants of the children’s levels of likeability among peers (White & Kistner, 2011), no significant association between positively biased perceptions of peer acceptance and proactive aggression was found.

Furthermore, as evaluating how liked we are by others relies on the capacity to infer what others think about us, self‐perceptions of likeability might be related to the theory of mind skills, defined as abilities to understand the mental state of others. Studies investigating the link between bullying and theory of mind (ToM) skills found that early adolescents who bully others had no more difficulty than their nonbullying counterparts in these tasks (Gini, 2006), and boy “ringleader bullies” were even found to have higher ToM skills (Caravita, Di Blasio, & Salmivalli, 2010). Taken together, these studies seem to hint that overestimation of likeability is more likely in reactively aggressive than proactively aggressive youth and in those with poor ToM skills. As bullying in adolescence is more strongly associated with proactive than reactive aggression (Pouwels et al., 2016), and bullying perpetrators do not have impaired ToM skills (Caravita et al., 2010; Gini, 2006), a positive link between bullying and overestimation of one’s likeability may not be as likely as suggested by the studies not distinguishing between reactive and proactive types of aggression.

In the present study, we will test the effects of bullying in adolescence on the overestimation of one’s likeability. As it has been suggested that the positive link between aggression and overestimation of one’s social competence held only for disliked children (e.g., De Castro, Brendgen, Van Boxtel, Vitaro, & Schaepers, 2007), we will also examine whether these effects vary depending on adolescents’ actual levels of likeability.

### 1.2 The superiority of popularity hypothesis

It is also possible that lower likeability does not discourage bullying perpetrators from engaging in bullying because they are more interested in being popular than they are in being liked. In other words, they may endorse the Machiavellian view that it is “much safer to be feared than loved” (Machiavelli, 1513/1981). Numerous studies on peer bullying and aggression in adolescence support this suggestion: Research directly investigating status goals (i.e., self‐reported importance of being popular and importance of being liked), have shown that endorsement of the popularity goal was positively related to peer‐reported aggression (Dawes & Xie, 2014; Faris & Ennett, 2012) and to self‐reported relational aggression (Dumas, Davis, & Ellis, 2017; Li & Wright, 2014). Other studies have examined the effects of adolescents’ prioritizing of popularity over other personals goals (such as friendship, achievement, and romance) on their social behaviors. Cillessen, Mayeux, Ha, De Bruyn, and LaFontana (2014) found that prioritizing popularity predicted higher levels of aggression, controlling for actual popularity. Using the same measure of prioritizing popularity, Van den Broek et al. (2016) found a positive correlation between bullying and prioritizing popularity. Consistent with these findings is a body of research on the link between social goals and aggression. Endorsing agentic goals, which reflect a desire for status in relationships, positively predicts proactive aggression among adolescents (Ojanen, Grönroos, & Salmivalli, 2005; Olthof, Goossens, Vermande, Aleva, & Van der Meulen, 2011; Sijtsema et al., 2009) and increases in relational aggression over time (Ojanen & Findley‐Van Nostrand, 2014).

When examining links between aggression and likeability goals, a very different pattern emerges: The more adolescents endorse the social preference goal, the less likely they are to report engaging in overt and relational aggression (Li & Wright, 2014). In addition, endorsing communal goals, which capture the desire to be close to others, was found to be either unrelated to aggression (Ojanen et al., 2005), negatively correlated with aggression in adolescent boys (van Hazebroek, Olthof, & Goossens, 2017), or predictive of decreased physical aggression (Ojanen & Findley‐Van Nostrand, 2014).

Taken together, these findings indicate that bullying perpetrators appear to favor popularity rather than likeability. Several of these studies further show that the positive association between valuing popularity and engaging in aggressive behavior varies depending on whether adolescents are actually popular. Those who prioritize popularity or strongly endorse the popularity goal tend to be especially engaged in bullying if they are also perceived as popular by their peers (Cillessen et al., 2014; Dawes & Xie, 2014). According to the studies by van den Broek et al. (2016) and Duffy et al. (2017), gender also plays a role. The finding that combining prioritizing popularity with high popularity predicted the highest levels of bullying held for adolescent boys only. In the present study, we expect that high engagement in bullying will be associated with favoring popularity over likeability, and we predict that this positive association will be stronger for more popular adolescents. Moreover, we will test whether these effects are further moderated by gender.

### 1.3 The unreachability of likeability hypothesis

A third possible explanation for why lower likeability does not prevent bullying perpetrators from engaging in the behavior is that they may believe they have no likeability to lose. They might assume that the peers who dislike them would not have liked them more had they not engaged in bullying. Whereas bullying perpetrators might believe that they have the capacity to achieve high popularity among peers, they might also think (correctly or not) that they would never be liked whether they bully or not. If this hypothesis were true, they would feel that they can only benefit by bullying others (by increasing their popularity), since they would have nothing to lose in terms of likeability. Therefore, it would be logical that low likeability as a side effect of bullying does not prevent them from bullying, as they feel they already are not well‐liked. When Machiavelli wrote that it is safer to be feared than to be loved, he specified “if one cannot be both.” Strangely, the idea that adolescent bullying perpetrators may believe that perceived popularity is within their reach, but likeability is out of their reach has not yet been considered in the scientific literature on bullying (or aggression) or in the literature on peer status. Evidence for this explanation would provide new avenues for interventions aimed at changing the cognitions of youth engaging in bullying.

In the literature, the studies that come closest to the current question regarding bullying/aggressive youth’s peer‐focused social cognitions are studies that examine the link between a hostile attribution bias and aggression. The hostile attribution bias is defined as the tendency to interpret others’ behavior as having hostile intent in ambiguous social situations, and according to the meta‐analysis by De Castro, Veerman, Koops, Bosch, and Monshouwer (2002), aggressive children are generally more likely to exhibit it. However, there is some indication that this positive association may hold only for reactively aggressive children, and not for children engaging in proactive aggression (Crick & Dodge, 1996; Dodge & Coie, 1987; Martinelli, Ackermann, Bernhard, Freitag, & Schwenck, 2018; Schwartz et al., 1998). Consistent with these early findings, studies comparing perpetrators, targets, and “bully‐victims” have shown that, when presented with ambiguous scenarios, “bully‐victims”—but not those who perpetrate bullying without being victimized themselves—were more likely than other peers to attribute hostility to the perpetrators, in childhood (Camodeca, Goossens, Schuengel, & Terwogt, 2003; Pouwels, Scholte, van Noorden, & Cillessen, 2015) and in adolescence (Guy, Lee, & Wolke, 2017).

Research on the link between aggression or bullying and hostile attribution bias offers precious information regarding the social cognition of perpetrators of bullying. However, attributing a harmful intent to other people’s behavior in ambiguous situations is different from assuming that people would dislike oneself regardless of whether one bullies or not. To our knowledge, no study has yet captured this type of cognition. In the present study, participants are presented with hypothetical bullying scenarios and asked to evaluate the extent to which the target and other classmates (in the story) would have disliked the bullying protagonist before any bullying started. We expected that adolescents who score higher on bullying engagement themselves would be more likely than others to endorse this belief.

## 2 STUDY 1: TESTING THE INACCURACY OF SELF‐PERCEIVED LIKEABILITY AND THE SUPERIORITY OF POPULARITY HYPOTHESES

The objective of this first study was to try and determine why being disliked would not prevent adolescent bullying perpetrators from engaging in bullying behavior, by testing two hypotheses. First, to examine whether bullying is associated with not being aware of being disliked (the *inaccuracy of self‐perceived likeability* hypothesis), we test the effects of bullying on an overestimation of one’s likeability. We further test if this effect is moderated by actual likeability. No specific expectation is formulated due to inconsistencies in previous research. Second, to examine whether bullying is associated with endorsing popularity goals more strongly than likeability goals (the *superiority of popularity* hypothesis), we test the effects of bullying on the difference between the importance attached to popularity and the importance attached to likeability. We expect this effect to be positive. In addition, we test whether this effect is moderated by adolescents’ peer status (popularity and likeability) and whether this moderating effect is further qualified by gender. In all analyses, we controlled for victimization, so as to capture the cognitions of those who bully but are not also being victimized.

### 2.1 Method

#### 2.1.1 Participants

The data used in the present study were part of a Dutch larger research project on children at risk for social and emotional problems, the Kandinsky Longitudinal Study. This project involves annual assessments of pupils in Grades 7–10, which are the first 4 years of secondary school in the Netherlands. There were 1,035 participants (495 boys and 540 girls). Their age ranged from 11.43 to 17.8 (*M* = 14.15; *SD* = 1.26). Ninety‐five percent of the participants were born in the Netherlands.

#### 2.1.2 Procedure

The parental consent procedure was under the responsibility of the school principals. Permission from parents was requested at the beginning of the school year for all tests/surveys deemed beneficial to the students. Schools sent parents a letter that included a description of the objective and procedure of the assessment, and a request to respond if they wanted their child not to participate. None of the parents expressed disapproval of the participation of their child. In addition, adolescents were asked to give their assent at the beginning of the assessment. None of them declined to participate, nor decided to opt out during or after the assessment. The data used by the researchers were anonymous.

Questionnaires were completed during regular teaching hours with the use of individual netbook computers. Right before the participants started to answer the questions, a survey administrator explained the goal of the study and informed participants of the anonymous processing and confidentiality of the data. Adolescents were told that they could stop participating at any time, had to answer the questions as honestly as possible and should not share these answers with others. Computerized peer nominations were used to assess adolescents’ peer status, bullying behavior, and victimization. For each nomination question, participants were presented with the full list of their classmates’ names and the number of nominations was unlimited (with a minimum of one). Self‐nominations were not possible, as the participant’s own name did not appear on the screen. Nominating a peer was done by clicking on his or her name.

#### 2.1.3 Measures

Bullying and victimization were assessed with peer nominations. Peer‐reported bullying was assessed with the item *Who bullies others?* and peer‐reported victimization with the item *Who is being bullied by others*? The total number of received nominations for each item was divided by the number of participants within each classroom to obtain proportion scores.

The two types of peer status—likeability and perceived popularity—were both measured with peer ratings. Participants were asked to rate the extent to which they liked each classmate and the extent to which they found each classmate popular on a 6‐point scale from *not at all* to *very much*. To obtain a score of likeability and popularity for each individual, received ratings were averaged per type of status per participant. Self‐perceived likeability was assessed by asking participants “*How much do your classmates like you?”* on a 6‐point scale from *not at all* to *very much*. An “overestimation of likeability” variable was computed by subtracting the self‐perceived likeability score from the average rating on likeability received from all classmates.

Importance of likeability and importance of popularity were each measured with one question; *How important is it for you to be liked by your classmates?* and *How important is it for you to be popular among your classmates?*, respectively. The questions again could be answered on a 6‐point scale from *not at all* to *very much*. The variable “favoring perceived popularity over likeability” was computed by subtracting the importance attached to likeability from the importance attached to popularity.

### 2.2 Results

#### 2.2.1 Do bullies overestimate their likeability?

Descriptive statistics and correlations for the main study variables are presented in Table 1. To examine whether bullying was significantly associated with overestimation of likeability, we ran a first regression analysis testing for the main effects of age, gender, actual (i.e., peer‐perceived) likeability, bullying, and victimization (Model 1) on an overestimation of likeability. All continuous variables were mean‐centered. The model was significant, *F*(5, 1004) = 56.11, *p* < 0.001, explaining 22% of the variance (Table 2). There was a significant effect of gender: Boys were more likely than girls to overestimate their likeability, *p* = 0.003. In addition, adolescents were more likely to overestimate their own likeability when they were younger, *p* = 0.004, lower in actual likeability, *p* < 0.001, and lower in victimization, *p* < 0.001. However, there was no significant effect of bullying, *p* = 0.604, suggesting that adolescents higher in bullying were not more likely than others to overestimate their likeability.

**Table 1:** Means, standard deviations, and correlations for the main study variables in the first sample (*N* = 1,092)

	*M* (*SD*)	1	2	3	4	5	6	7	8
1. Peer‐perceived popularity	4.02 (0.83)	–							
2. Peer‐perceived likeability	4.30 (0.53)	0.60***	–						
3. Self‐perceived likeability	4.56 (0.75)	0.23***	0.24***	–					
4. Overestimation of likeability	0.27 (0.81)	−0.18***	−0.44***	0.77***	–				
5. Importance of likeability	4.70 (1.11)	0.11***	0.09**	0.26***	0.18***	–			
6. Importance of popularity	3.69 (1.23)	27***	0.15***	0.23***	0.11***	0.45***	–		
7. Favoring popularity	−1.01 (1.23)	0.17***	0.07*	−0.01	−0.05	−0.45***	0.59***	–	
8. Bullying	0.08 (0.13)	0.26***	−0.27***	−0.06	0.12***	−0.05	0.10**	0.14***	–
9. Victimization	0.07 (0.15)	−0.54***	−0.48***	−0.23***	0.10**	−0.09**	−0.11**	−0.03	0.08*

* *p* < 0.05.

** *p* < 0.01.

*** *p* < 0.001.

**Table 2:** Regression analyses predicting adolescents’ overestimation of their own likeability (*N* = 1,092)

	Model 1: Main effects			Model 2: Interaction effects		
	*B*	*SE*	*β*	*B*	*SE*	*β*
Intercept	0.338***	0.033		0.342***	0.033	
Age	−0.055**	0.019	−0.085	−0.055**	0.019	−0.086
Gender (girl)	−0.138**	0.047	−0.085	−0.133**	0.047	−0.083
Bullying	−0.100	0.193	−0.016	0.039	0.239	0.006
Peer‐perceived likeability	−0.727***	0.054	−0.476	−0.728***	0.054	−0.476
Victimization	−0.697***	0.172	−0.132	−0.700***	0.172	−0.132
Bullying*peer‐perceived likeability				0.347	0.351	0.035
*R* ^2^	0.22			0.22		

*Note*. The effects of bullying remain nonsignificant even when victimization is not controlled for.

* *p* < 0.05.

** *p* < 0.01.

*** *p* < 0.001.

A second model was run (Model 2), which included the interaction between bullying and actual likeability in addition to all the predictors of the first model. This model was significant, *F*(6, 1003) = 46.92, *p* < 0.001, explaining 22% of the variance (Table 2). The interaction between bullying and actual likeability was not significant, *p* = 0.322, suggesting that the effects of bullying on an overestimation of one’s likeability are not moderated by adolescents’ actual likeability.

#### 2.2.2 Do bullying perpetrators favor perceived popularity over likeability?

To test whether those higher in bullying are more likely to favor popularity over likeability, we first tested for the main effects of age, gender, bullying, and victimization on favoring popularity over likeability in a regression analysis. All continuous variables were mean‐centered. The model, presented in Table 3, was significant, *F*(4, 1005) = 12.25, *p* < 0.001, explaining 5% of the variance in predicting favoring popularity over likeability. There was a positive effect of age, *p* < 0.001; gender, *p* = 0.001; and bullying, *p* < 0.001, but no significant effect of victimization, *p* = 0.179. Adolescents higher in bullying, boys, and older adolescents were more likely to favor popularity over likeability.

**Table 3:** Regression analyses predicting favoring popularity over likeability (*N* = 1,092)

	Main effects			Interaction with popularity			Interaction with likeability		
	*B*	*SE*	*β*	*B*	*SE*	*β*	*B*	*SE*	*β*
Intercept	−0.867***	0.055		−0.862***	0.056		−0.862***	0.056	
Age	0.109***	0.030	0.111	0.083**	0.031	0.084	0.085**	0.032	0.087
Gender (girl)	−0.272**	0.078	−0.111	−0.256**	0.079	−0.104	−0.257**	0.079	−0.105
Bullying	1.102***	0.310	0.113	0.891*	0.362	0.092	1.601***	0.400	0.165
Victimization	−0.334	0.249	−0.042	0.379	0.309	0.047	−0.034	0.288	−0.004
Peer‐perceived popularity				0.236***	0.059	0.160			
Peer‐perceived likeability							0.186*	0.090	0.080
Bullying × Peer‐perceived popularity				−0.406	0.348	−0.040			
Bullying × Peer‐perceived likeability							0.750	0.587	0.050
*R* ^2^	0.05			0.06			0.05		

*Note*. The two way interaction between bullying and gender, as well as three‐way interactions between bullying, gender and each type of status were nonsignificant and are not included in the models above.

* *p* < 0.05.

** *p* < 0.01.

*** *p* < 0.001.

To examine whether the association between bullying and favoring popularity would be moderated by adolescents’ popularity or likeability, we ran two additional models including the main effect of popularity and the interaction between popularity and bullying (the “popularity” model) and the main effect of likeability and the interaction between likeability and bullying (the “likeability” model). Models were conducted separately for popularity and likeability to prevent multicollinearity, since the correlation between the two types of status was high (*r* = 0.60, *p* < 0.001).

The “popularity” model was significant, *F*(6, 981) = 10.85, *p* < 0.001, explaining 6% of the variance. There was a positive main effect of popularity; higher popularity predicted a stronger tendency to favor popularity over likeability, *p* < 0.001. In this model including the effects of perceived popularity, bullying remained a significant positive predictor, *p* = 0.014. The effects of bullying, however, did not significantly vary depending on adolescents’ levels of popularity, *p* = 0.243. The “likeability” model was significant, *F*(6, 1003) = 9.20, *p* < 0.001, explaining 5% of the variance. There was a positive main effect of likeability, *p* = 0.039, but no significant interaction between bullying and likeability in the prediction of favoring popularity over likeability, *p* = 0.202. In this model controlling for likeability, the effects of bullying remained significant, *p* < 0.001. It should be noted that when popularity and likeability are entered in the same model, only popularity has a significant effect on favoring popularity over likeability.

## 3 STUDY 2: THE UNREACHABILITY OF LIKEABILITY HYPOTHESIS

The goal of the second study was to examine whether bullying perpetrators might believe that they have no likeability to lose, which would explain why low likeability does not deter them from bullying. To put this explanation to the test, we exposed participants to vignettes of hypothetical bullying scenarios followed by questions regarding the target’s and other peers’ disliking of the bullying protagonist in the story. Specifically, participants were first asked to which extent they believed the target (Question 1) and classmates (Question 2) disliked the bullying perpetrator. In addition, they were asked to assess the extent to which they believed the target (Question 3) and classmates (Question 4) disliked the bullying perpetrators before the bullying behavior started. We expected that adolescents higher in bullying would be more likely to endorse beliefs that targets and classmates would have disliked the bullying perpetrator before the bullying started.

### 3.1 Method

#### 3.1.1 Participants

The sample for this study consisted of 693 adolescents (51.2% boys) from the first three grades of secondary school. They belonged to 29 classrooms in 15 schools in the Netherlands. Their age ranged from 11‐ to 17‐years old (*M* = 12.92, *SD* = 0.86) and 96.1% were born in the Netherlands. Among the 693 adolescents, 607 received parental consent to participate (87.6%) and among those, six adolescents did not give their assent to participate in the study, resulting in 601 participants. The mean age, gender distribution, and origin in the participating sample were the same as in the larger sample.

#### 3.1.2 Procedure

The participants were recruited by contacting individual teachers from 15 secondary schools in various cities across the Netherlands. The data were collected as part of a larger study designed to test the effectiveness of a small intervention. It consisted of three assessments (two preintervention and one postintervention). Only the data collected at the two preintervention assessments, which took place about 1 week apart, were used in the current report: Measures of peer status and vignette questions designed to assess beliefs regarding likeability were collected at the first data collection moment, and measures of aggression and victimization were collected at the second data collection moment (i.e., these measures were thus not assessed longitudinally). Active parental consent was used: About 2 weeks before data collection, parents received a letter describing the general objective of the study, and a request to return the signed form if they gave permission to their child to participate. On the first day of data collection, adolescents who had received parental consent were asked to give their assent before survey administration. Data collection took place during regular school hours. It was conducted by university undergraduate assistants in the presence of the participants’ teacher. Participants were informed that they could stop participating at any time and the data would remain anonymous (only code numbers—and no names—were used to enter the data). During data collection, nonparticipants were given a general knowledge questionnaire to complete to preserve the anonymity of their nonparticipation.

#### 3.1.3 Measures

The data used in the current study consisted of peer nominations for bullying and victimization, as well as self‐reports of beliefs regarding hypothetical bullies’ degree of likeability. For the peer nominations, participants were presented with the list of their classmates and were asked to nominate the ones who fitted the description. The number of classmates they could nominate was unlimited and they were allowed to nominate none. The total number of received nominations for each item was divided by the number of participants within each classroom to obtain proportion scores. Peer‐reported bullying was assessed with three items capturing three types of aggression: physical (*Who kicked, pushed, or hit another student at school in the past week?*), verbal (*Who called another student names, or said mean things to another student at school in the past week?*), and relational (“*Who spread rumors or lies about another student, or excluded another student from the group at school in the past week*?”). Proportion scores for the three items were averaged to create a composite bullying score. Peer‐reported victimization was assessed in the same way: The proportion scores for three items, capturing physical, verbal, and relational victimization were averaged to create a composite victimization score.

Participants’ beliefs about the likeability of bullying perpetrators were measured by using three vignettes, each describing a hypothetical bullying incident (one vignette per type of aggression: physical, verbal, and relational) involving one victimized student and one bullying student. These vignettes were adapted from vignettes used by Yoon (2004)—who had adapted them from Craig, Henderson, and Murphy (2000)—and translated in Dutch (see appendix for the English version). After reading each vignette, the participants were asked a series of four questions: *According to you, how likely is it that the victim dislikes the bully* (Q1), *the classmates dislike the bully* (Q2), *the victim disliked the bully before the bullying started* (Q3), *the classmates disliked the bully before the bullying started* (Q4). Ratings were given on a 7‐point scale (from 0 = *very unlikely* to 6 = *very likely*). As the same questions were asked for each vignette, a score for each question was computed by averaging the ratings for that question across the three vignettes. The reliability coefficients for each question were as follows: *α* = 0.68 for Q1, *α* = 0.67 for Q2, *α* = 0.72 for Q3, and α = 0.73 for Q4.

### 3.2 Results

The descriptive statistics and correlations for all variables are presented in Table 4. To test the hypothesis that adolescents higher in bullying would be more likely to endorse beliefs that victimized students and classmates would have disliked the bullying perpetrator before any bullying started, we ran four regression models. In each model, we tested the effects of bullying on answers to each of the four vignette questions, controlling for age, gender, and victimization. We were particularly interested in the effects of bullying on answers to Questions 3 and 4, but also investigated answers to Questions 1 and 2 for comparison purposes. Results are shown in Table 5.

**Table 4:** Descriptive statistics and correlations among Study 2 variables (*N* = 601)

	*M* (*SD*)	Range	2	3	4	5	6
1. Bullying	0.03 (0.05)	0–0.38	0.68***	0.06	0.03	0.07	0.13**
2. Victimization	0.03 (0.04)	0–0.42	–	0.05	−0.02	0.01	0.07
3. Q1: *How likely is it that the victim dislikes the bully?*	4.45 (1.37)	0–6		–	0.24***	0.26***	0.12**
4. Q2: *How likely is it that others in the class dislike the bully?*	2.94 (1.36)	0–6			–	0.17***	0.53***
5. Q3: *How likely is it that the victim would have disliked the bully before any bullying started?*	2.83 (1.57)	0–6				–	0.49***
6. Q4: *How likely is it that others would have disliked the bully before the bullying started?*	2.27 (1.35)	0–6					–

** *p* < 0.01.

*** *p* < 0.001.

**Table 5:** Standardized coefficients for four regression models predicting answers to the four vignette questions (*N* = 601)

	How likely is it that the victim dislikes the bully?	How likely is it that others in the class dislike the bully?	How likely is it that the victim would have disliked the bully before the bullying started?	How likely is it that others would have disliked the bully before the bullying started?
Age	−0.018	0.138**	−0.006	0.096*
Gender	−0.075	−0.037	0.011	−0.051
Bullying	0.041	0.093	0.118*	0.145*
Victimization	0.009	−0.095	−0.076	−0.047
*F*	1.27	3.50**	1.00	3.81**
*R* ^2^	0.01	0.03	0.01	0.03

* *p* < 0.05.

** *p* < 0.01.

The model for Q1 was overall not significant, *F*(4, 524) = 1.27, *p* = 0.281, and neither bullying, *p* = 0.495, nor age, *p* = 0.675, gender, *p* = 0.096, or victimization, *p* = 0.881 had a significant effect on the endorsement of beliefs that the target in the vignette dislikes the bullying perpetrator. The model for Q2 was overall significant, *F*(4, 524) = 3.50, *p* = 0.008. Among the individual predictors, only age had a significant effect on the endorsement of beliefs that hypothetical classmates of the bullying perpetrator and target in the vignette would dislike the bullying perpetrator, *p* = 0.002. Older adolescents were more likely than younger adolescents to think that the bullying perpetrator would be disliked by classmates. However, there was no significant effect of bullying, *p* = 0.117; gender, *p* = 0.408; or victimization, *p* = 0.101.

The model for Q3 was not significant overall, *F*(4, 524) = 1.00, *p* = 0.406. Nevertheless, bullying had a significant positive effect on the endorsement of the belief that the victimized student would have disliked the bullying perpetrator even before any bullying started, *p* = 0.048. Neither age, *p* = 0.894; gender, *p* = 0.804; or victimization, *p* = 0.197 were significant predictors. The model for Q4 was overall significant, *F*(4, 524) = 3.81, *p* = 0.005. Both age, *p* = 0.026, and bullying, *p* = 0.014, were positively and significantly associated with endorsement of beliefs that classmates would have disliked the bullying perpetrator even before any bullying started. There was no significant effect of gender, *p* = 0.249 or victimization, *p* = 0.421.

## 4 DISCUSSION

Understanding the psychological mechanisms underlying bullying perpetration among youth is essential for effective antibullying intervention efforts. That bullying tends to be rewarded with high perceived popularity in adolescence is now widely acknowledged as a key explanation for adolescents’ engagement in bullying (e.g., Pouwels et al., 2016; Reijntjes et al., 2013a, 2013b; Vaillancourt et al., 2003). However, research also suggests that bullying is associated with losses in likeability (e.g., Pouwels et al., 2016). Our aim was to elucidate why such a cost in terms of likeability does not deter bullying perpetrators from engaging in bullying behavior. Three possible explanations were put to the test across two studies. In Study 1, we investigated the possibility that adolescent bullying perpetrators may overestimate their likeability (the *inaccuracy of self‐perceived likeability* hypothesis), and the possibility that decreased likeability may not matter to them because they value being popular more than they value being liked (The *superiority of popularity* hypothesis). In Study 2, we explored the possibility that adolescents who bully others might think that they would not be liked whether they bully or not and therefore have no likeability to lose by bullying (the *unreachability of likeability* hypothesis).

### 4.1 Support for two of the three explanations

No support was found for the *inaccuracy of self‐perceived likeability* hypothesis: While victimized adolescents had a tendency to underestimate their likeability, adolescent bullying perpetrators were no more likely than their peers to overestimate how much they were liked by their classmates, regardless of their own likeability. Adolescents’ accuracy at evaluating their own likeability was thus not related to their bullying behavior. To our knowledge, our study is the first to examine this question with regard to bullying specifically rather than aggressive behavior in general. Indeed, our finding does not reflect the typical finding in research linking aggressive behavior to self‐enhancing tendencies in children (e.g., De Castro et al., 2007). Such links, however, are more likely to be observed when examining reactive aggression. In fact, children with ADHD, who tend to use reactive rather than proactive aggression (Murray, Obsuth, Zirk‐Sadowski, Ribeaud, & Eisner, 2016), also tend to overestimate their social acceptance (Hoza, Pelham, Dobbs, Owens, & Pillow, 2002). Our results are consistent with studies showing that those who engage in bullying or proactive aggression, do not lack the ability to understand the mental state of others (Caravita et al., 2010; Gini, 2006). They indicate that youth’s engagement in bullying cannot be attributed to their blindness for their (low) likeability level among their classmates.

The results of the current study show that bullying others is positively associated with a tendency to value being popular more than being liked. This finding adds to the increasing number of studies showing that aggressive youth aim for high popularity among peers but are not necessarily concerned with being liked (e.g., Li & Wright, 2014) by showing a similar effect for bullying in adolescence. In addition, results indicated that being popular was associated with valuing popularity more strongly than likeability. Although some previous studies showed that actual status and bullying interacted in predicting boys’ prioritizing of popularity (Duffy et al., 2017; Van den Broek et al., 2016), the current study did not find such moderation effects. One strength of our study was to combine ratings of the importance of being popular and the importance of being liked in a single measure, quantifying the degree to which one was more strongly pursued compared to the other. To our knowledge, this is the first study to quantify the prioritizing of popularity over likeability so precisely.

The most likely explanation for the finding that bullying perpetrators prefer popularity is that it is associated with greater social power (Vaillancourt et al., 2003). This explanation is consistent with an evolutionary approach. Proactively aggressive youth’s preference for popularity may also reflect their Machiavellian approach to social interactions, according to which individuals find it safer to be feared when faced with choosing between being loved and being feared (Machiavelli, 1513/1981). However, several points remain to be clarified in future research: Although the desire for interpersonal attachment may be a fundamental human need (Baumeister & Leary, 1995), it is possible that bullying perpetrators value having the social connections that perceived popularity provides but are indifferent to the quality or depth of these relationships. Moreover, it is unknown whether this higher value attached to popularity is a stable personality trait present early in life or whether it is acquired and can be potentially unlearned.

The current set of studies took the exploration of bullying students’ cognitions and motivations in relation to peer status a step further. Support was found for the hypothesis that bullying perpetrators might believe that targets of bullying and other peers would not like them anyway, whether they engage in bullying or not. When exposed to hypothetical bullying scenarios, adolescents higher in bullying were not more likely than others to think that the hypothetical target and classmates disliked the bullying protagonist. However, they were more likely than others to report thinking that the target and the classmates would have disliked the bullying protagonist even before any bullying started. This implies that adolescent bullying perpetrators may not be deterred by the costs of bullying in terms of likeability possibly because the perceived costs are relatively low. They might think that their likeability will not decrease much when they engage in bullying, as they already think that they are being disliked. What remains to be determined is whether the fact that bullying perpetrators find popularity more important than likeability might be a consequence of their perception of likeability as being out of reach. The reasons why bullies tend to prefer popularity are still unclear. Our finding that they may believe they have no likeability to lose provides a new, testable explanation and sheds light on a cognitive process that can potentially be modified by intervention. Another venue for further exploring this type of reasoning in bullying perpetrators would be to assess cognitions such as “*Regardless of my behavior, I will always be disliked”* more directly and more personally related, instead of having them judge others in a hypothetical situation. Ideally, youth whose cognitions and bullying behavior are measured would have to be followed longitudinally to examine whether this “likeability is unreachable” cognitions actually are predictive of increases in bullying engagement over time.

### 4.2 Limitations and future research

As expected, in the data we analyzed the correlation between bullying and peer‐perceived likeability was negative (*r* = −0.27). Nevertheless, an important limitation of our study is that with these data we cannot provide evidence that bullying results in a loss of likeability over time. Despite multiple cross‐sectional studies showing that adolescent bullying perpetrators tend to be disliked (e.g., Dijkstra et al., 2008; Pouwels et al., 2016; Vaillancourt et al., 2003) and longitudinal investigations of the effects of likeability on future bullying behavior (Sentse et al., 2014), the current literature is still lacking in longitudinal research on the link between bullying and changes in likeability over time. One exception is the study by Reijntjes et al. (2013a) which examined prospective links between bullying and social acceptance using joint trajectories analyses. Whereas they found that boys high in bullying were more likely than other boys to be disliked, there was no clear indication that bullying was associated with decreases in likeability over time. Further empirical tests of this longitudinal association are needed.

Furthermore, the results for the second and third hypotheses should be interpreted with caution. First, the effect sizes are very small, suggesting that these effects may be difficult to detect with smaller samples. Moreover, regarding the second hypothesis, we did not control for variables which have been shown to be positively associated with prioritizing popularity, such as substance use and sexual activity (Van den Broek et al., 2016) or having friends who prioritize popularity (Faris & Ennett, 2012). Second, we cannot be certain that the questions asked in Study 2 about the victimized student and other classmates’ liking of the bullying protagonist *before the bullying started* were interpreted as intended (i.e., before any bullying happened at all). It is a strong possibility that participants interpreted the question as disliking the bullying perpetrator before this particular incident began. Youth higher in bullying thus expected already lower likeability of the hypothetical bullying protagonist before the described incident. However, with the current measure, it is difficult to determine exactly how far back this “prior liking” refers to. Although this ambiguity does not invalidate our findings—as the alternative interpretation of the question does not explain why youth higher in bullying would differ in their expectations from other adolescents—it will be important to use a less ambiguous formulation in replication attempts of these findings. Third, it should be noted that the measure of bullying used in Study 2 tapped into three forms of intentional aggression but did not capture the power differential and repetition that characterize bullying. This means that our measure may have overestimated the prevalence of bullying perpetrators since adolescents who behaved aggressively once or were merely involved in a conflict could have scored high on that variable. Our measure also did not distinguish proactive aggression from reactive aggression, although they differ in important ways. Unlike proactive aggressors, reactive aggressors tend to be unpopular (Stoltz, Cillessen, van den Berg, & Gommans, 2016) and prone to hostile attribution bias (Martinelli et al., 2018). Reactive aggressors may, therefore, be more likely to endorse the belief that bullying perpetrators would be disliked regardless of their behavior. As we controlled for victimization levels, effects of bullying in our analyses do tend to test the effects of more proactive types of aggression. For this reason, we do not expect that our results would have been very different with a more specific assessment of bullying; however, future investigations of this hypothesis should clarify whether the effects differ for (victimization controlled) general aggression versus bullying.

The use of vignettes of hypothetical bullying scenarios to assess beliefs regarding the likeability of bullying students had the advantage of possibly inhibiting socially desirable responding: Perpetrators of bullying may be more honest when reporting beliefs about imaginary characters than they would be when having to report their actual cognition. Nonetheless, we cannot be certain that their reported beliefs regarding the likeability of the protagonists in the bullying scenarios accurately reflect their beliefs about their own likeability. To test more directly whether bullying adolescents believe that victimized students and/or other classmates would have disliked them even before they started engaging in bullying, dyadic analyses should be utilized: Asking participants “*Who do you think likes/dislikes you at the moment?*” and “*Who do you think liked/disliked you already the first time they met you?*” and combining this information with data on who bullies whom would shed light on whether bullying perpetrators (a) are more likely than others to assume that the peers who they think dislike them would dislike them regardless of their behavior and (b) are more likely to make this assumption of immediate dislike for the peers they victimize compared with the peers they do not target.

Moreover, we have not examined by which type of peers those who bully thought they were disliked and by which type of peers they were actually disliked (e.g., friends vs. nonfriends and victims vs. nonvictims), as our measure of actual likeability resulted from averaging ratings across peers, and our measure of self‐perceived likeability only considered general likeability among peers. However, research suggests that the rejection of bullying perpetrators may not be equally shared among their classmates, but is restricted to their targets (Hafen, Laursen, Nurmi, & Salmela‐Aro, 2013) and to peers for whom they represent a threat (e.g., Veenstra, Lindenberg, Munniksma, & Dijkstra, 2010). To more accurately test whether bullying perpetrators are aware of being disliked (first hypothesis), future studies should include dyadic analyses to investigate whether they report being disliked by the specific peers who actually report disliking them. Similarly, our global measure of reported importance of being liked did not enable us to capture by whom participants found it important or not to be liked. It is conceivable that students who bully do not find it important to be liked by some others, their targets in particular, but still care about being liked by a group of friends for instance. Using more specific questions, such as “*I only find it important that my friends like me, whether other classmates like me or not is not important to me*” would provide useful information.

Finally, the current study used an ethics committee‐approved passive consent procedure and had nonparticipating students still on the nomination roster. A benefit of this procedure is a high participation rate, which increases the reliability and validity of the peer nominations. It also protected the nonparticipants from identification by their participating classmates. However, passive consent is not the most ethically rigorous approach, as classmates could nominate nonparticipating classmates and, therefore, some information about nonparticipants was initially available to the researchers.

## 5 CONCLUSIONS

Achieving a significant breakthrough in the fight against school bullying calls for a more precise understanding of the cognitions that underlie youth’s decisions to instigate bullying against peers. By focusing on the construct of perceived popularity, the literature of the past two decades has emphasized the importance of status rewards in explaining aggression and bullying behavior in adolescence. The present set of studies provides new insight into the role of peer status in perpetrators’ decision to engage in bullying by examining the role of three different types of cognitions related to likeability among peers. It demonstrates that the behavior of bullying perpetrators cannot be attributed to their lack of awareness of their own likeability, and thereby sheds light on the mixed body of literature on aggressive adolescents’ self‐perceptions. Using a new measure that quantifies the degree to which one type of status (popularity or likeability) is considered more important than the other, it even more strongly corroborates previous findings that proactively aggressive youth strongly value popularity but not likeability. Moreover, the current study demonstrates these patterns for bullying specifically, whereas most of the previous work on accuracy of perception of likeability and the importance of popularity and likeability has focused on aggression more generally, most distinguishing in the nature of (overt vs. relational) or motivation for (proactive vs. reactive) aggression.

Furthermore, our results suggest that bullying perpetrators may be apt to believe that likeability is out of their reach. This possibility has, to our knowledge, never been examined before, and might add to our ability to explain why lower likeability does not deter some youth from engaging in bullying. Should this finding be replicated with more precise dyadic analyses in future studies, it could open new doors for antibullying intervention. Addressing beliefs that likeability is not reachable and unaffected by bullying might help in preventing or reducing bullying.

## Supporting information

Supporting information
