# Supplementary material for: Why does decreased likeability not deter adolescent bullying perpetrators?
Source: Aggress Behav. 2019 Feb 1;45(3):348–59. doi: 10.1002/ab.21824 (PMC6590429; doi:10.1002/ab.21824)
Supplement: Supplementary file 1 — Supporting information [file AB-45-348-s001.docx]

Appendix

Vignettes of Hypothetical Bullying Incidents for Three Types of Bullying

Physical bullying

During class, students are working in groups of four on a group assignment. While the students are getting in their group, Bram pushes Jan with enough force so that he falls on the floor. The push was clearly intentional and was not provoked. Jan yells “Stop pushing me around, just go away!” This kind of thing happened already many times before between Bram and Jan.

According to you, how likely is it that

1. Jan dislikes Bram?
2. other students in the class dislike Bram?
3. Jan would have disliked Bram even before this pushing started?
4. other students in the class would have disliked Bram even before this pushing started?

Relational bullying

The teacher is asking pupils to form groups to work together on a project. Sanne is sitting close to Emma and two other girls. She tells the two other girls "Let's do it together" and completely ignores Emma. When Emma asks if she can join them, Sanne tells her "No, we don't want you in our group." Emma finds herself sitting alone and she has tears in her eyes. This kind of thing happened already many times before between Sanne and Emma.

According to you, how likely is it that

1. Emma dislikes Sanne?
2. other students in the class dislike Sanne?
3. Emma would have disliked Sanne even before this not allowing to join started?
4. other students in the class would have disliked Sanne even before this not allowing to join started?

Verbal bullying

In the hallway, Sam chants to Nick “Teacher’s pet, suck-up, kiss-ass.” Nick tries to ignore the remark but sulks at his desk. This happened already many times before between Sam and Nick.

According to you, how likely is it that

1. Nick dislikes Sam?
2. other students in the class dislike Sam?
3. Nick would have disliked Sam even before this name calling started?
4. other students in the class would have disliked Sam even before this name calling started?
